# Commentary on the article recently published in the journal by Marquito et. al.

**DOI:** 10.1590/2175-8239-JBN-2020-0129-LET

**Published:** 2020-11-16

**Authors:** Juliana Rodrigues da Silva Lopes, Hanna Mina dos Santos Correa, Cristina Salles

**Affiliations:** 1 Escola Bahiana de Medicina e Saúde Pública, Salvador, BA, Brazil.

Dear Editor,

First, we would like to congratulate the authors for the publication of the article "*Pharmacotherapy assessment in chronic kidney disease: validation of the pair instrument for use in Brazil*"^1^. It is undeniable the relevance of efforts like this for the validation of instruments, aiming at its use in our country.

However, we would like to consider that we feel data are lacking regarding the use of antidepressant medications in this population. The mental disorder most found in patients with chronic kidney disease^2^ is depression, usually justified by the high emotional burden that accompanies a chronic disease, associated with somatic symptoms, which interfere in the quality of life of these patients.

In addition to depression, sleep disorders associated with chronic kidney disease are also frequently reported in the literature. This problem is usually related to periodic limb movements during sleep, or obstructive sleep apnea, which can result in daytime sleepiness and other consequences including irritability, confusion, depression itself or even paranoia^3^. Therefore, effective management of sleep disorders in these patients is essential to reduce the high rate of morbidity and mortality^4^. This management may include the use of sleep-inducing medications, which were also not mentioned during the article.

Considering depression and sleep disorder as related comorbidities in the population studied, the drugs that are part of the treatment of these diseases continually make up the polypharmacy related to the treatment of chronic kidney patients. For this reason, I suggest that you evaluate these suggestions, as well as emphasizing medications for the treatment of cardiovascular and metabolic symptoms.

I conclude once again by thanking the authors for their initiative in carrying out a study that brings as a result a new instrument validated for use in Brazil, useful for those who work with these patients.
